# Elucidating therapeutic mechanisms of naringin and phloridzin on ulcerative colitis mice by metabolomics-based comparative analysis

**DOI:** 10.1007/s13659-026-00620-4

**Published:** 2026-06-01

**Authors:** Jing Yang, Chongxin Kang, Chulei Xiao, Yusi Zhang, Yihong Mai, Yinghong Huang, Yi Zhan, Hetong Liu, Xian Wang

**Affiliations:** Key Laboratory of Analytical Chemistry of the State Ethnic Affairs Commission, School of Chemistry and Materials Science, South-Central Minzu University, Wuhan, 430074 Hubei China

**Keywords:** Naringin, Phloridzin, Ulcerative colitis, Therapeutic mechanisms, Metabolomics, Tryptophan pathway

## Abstract

Naringin and phloridzin are structurally similar flavonoids with anti-inflammatory, antioxidant, and antimicrobial properties, showing considerable potential as natural therapeutic agents. This study demonstrated that naringin and phloridzin exert anti-inflammatory effects and preserve normal intestinal function in UC mice. This protective effect is mediated by inhibition of the NF-κB signalling pathway. To investigate metabolic differences, we employed UPLC-MS/MS-based widely targeted metabolomics alongside targeted analyses of tryptophan (Trp) and short-chain fatty acids (SCFAs). Comparative analysis of metabolic profiles, differential metabolites, and associated pathways revealed regulatory effects on Trp and SCFAs. However, naringin demonstrated superior regulation of Trp metabolism and SCFA levels compared to phloridzin, resulting in more pronounced improvement of gut homeostasis. These findings elucidate the metabolic mechanisms underlying both flavonoid treatments and underscore their potential as complementary therapies for UC.

**Graphical abstract:**

Naringin and phloridzin exert protective effects against DSS-induced ulcerative colitis by modulating tryptophan metabolism and suppressing inflammation through inhibition of the NF-κB signaling pathway

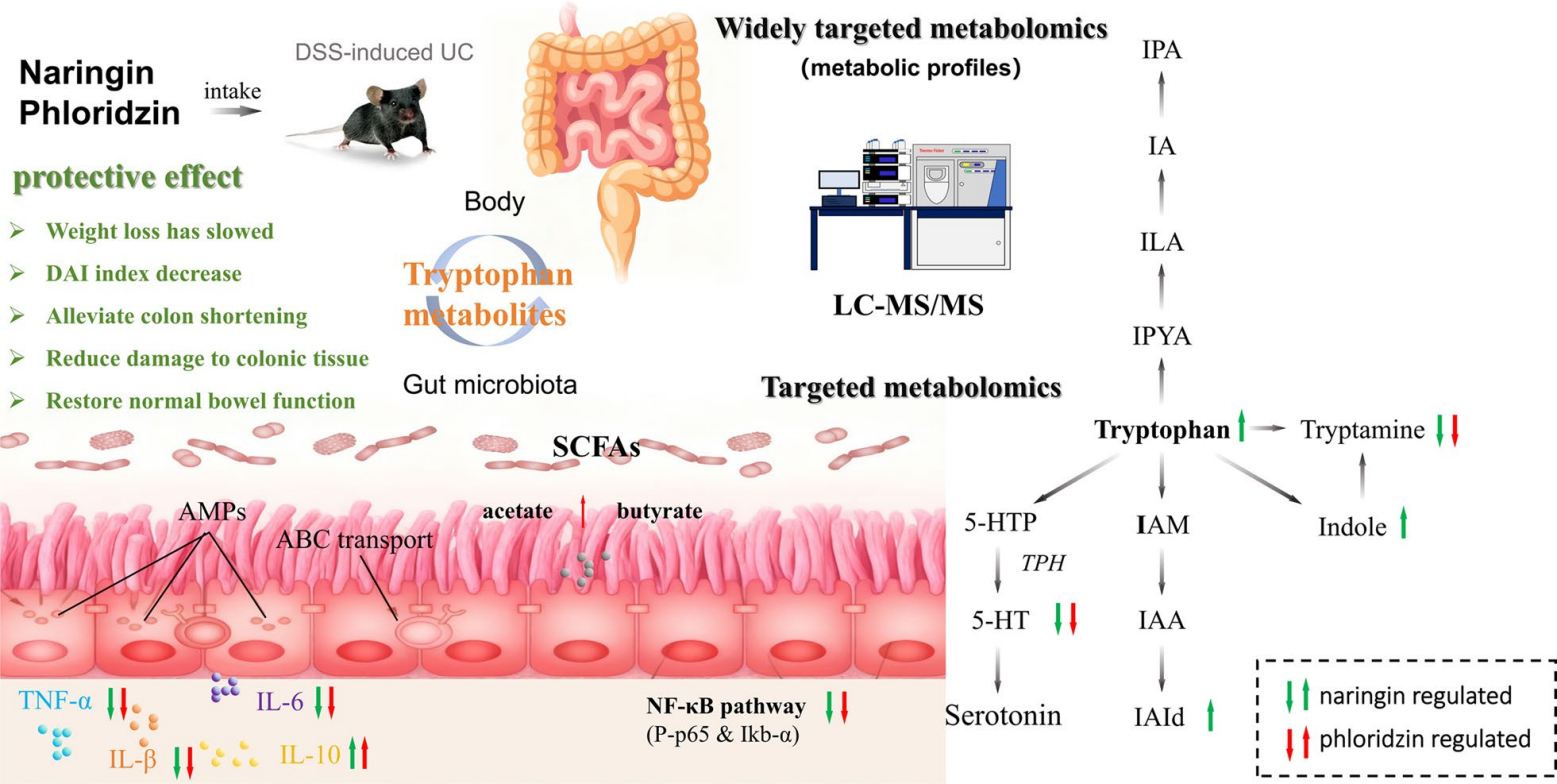

**Supplementary Information:**

The online version contains supplementary material available at 10.1007/s13659-026-00620-4.

## Highlights

1. Widely targeted and targeted metabolomic methodologies were applied to elucidate therapeutic mechanisms of naringin and phloridzin on mice DSS-induced colitis.
2. Both naringin and phloridzin exerted anti-inflammatory effects, whereas naringin exhibited a more pronounced protective effect on gut health through modulation of tryptophan metabolic channel.
3. Naringin and phloridzin demonstrated comparable therapeutic alleviation, highlighting the role of flavonoids in maintaining intestinal health.

**Supplementary Information:**

The online version contains supplementary material available at 10.1007/s13659-026-00620-4.

## Introduction

Ulcerative colitis (UC) is a chronic inflammatory disease predominantly affecting the colon [1], and together with Crohn’s disease (CD), it constitutes the two major subtypes of inflammatory bowel disease (IBD) [2]. The hallmark clinical manifestations of UC include urgency of defecation, bloody diarrhoea, and associated symptoms such as muscle weakness, vomiting, and weight loss [3]. Recent studies have provided substantial insights into the pathogenesis of UC. On one hand, UC is closely linked to dysfunction of colonic epithelial cells and impairment of the mucosal and epithelial barriers [4]; on the other hand, aberrant expression of peroxisome proliferator-activated receptor gamma (PPAR-γ) in colonic cells has been demonstrated to contribute to the disease’s pathological progression [5]. Although numerous drugs are currently available for the treatment of ulcerative colitis (UC), some patients exhibit poor responses to conventional therapies or develop drug resistance. Therefore, the exploration of novel therapeutic strategies and agents has become a critical focus in current research.

Natural products have attracted increasing attentions owing to their multi-target activities and low toxicity, among which flavonoids such as naringin and phloridzin from traditional Chinese medicine show remarkable pharmacological activities [6]. Naringin, a flavanone glycoside formed from the flavanone naringenin and the disaccharide neohesperidose, is responsible for the bitter taste of citrus fruits [7]. It has been widely demonstrated to possess diverse pharmacological properties, including antioxidant, anticancer [8], antiulcer, antibacterial, anti-inflammatory [9], hepatoprotective, antidiabetic [10], and hypolipidemic effects [11]. Recent studies have revealed that naringin can be utilized in the treatment of ischemic necrosis of skin flaps, metabolic syndrome, and has shown protective effects in ulcerative colitis. Phloridzin, also known as phloretin-2′-O-glucoside [12], belongs to the dihydrochalcone subclass of flavonoids and is primarily derived from the leaves, seeds, and other parts of apple trees. Phloridzin exhibits potent antioxidant and anti-inflammatory effects [13], along with hypoglycaemic [14] and hepatoprotective properties [15]. Research by Cao et al. indicated that naringin exerted a mitigating effect on enteritis and suggested that this was associated with the activation of PPARγ [16].. Although the pharmacological effects of naringin and phloridzin have been extensively studied, their therapeutic roles in UC and their underlying metabolic mechanisms remain incompletely understood. Particularly, research on the metabolic mechanisms of phloridzin remains scarce and is less extensive than that on naringin, and comparative investigations into their therapeutic efficacy in colitis treatment are still lacking.

As an integral branch of systems biology, metabolomics—together with proteomics, transcriptomics, and genomics—has demonstrated substantial potential in therapeutic applications, facilitating advances in diagnosis [17], treatment [18], and patient follow-up [19]. For example, metabolomics has demonstrated potential in identifying metabolic biomarkers specific to diabetic nephropathy [20]. A novel and more practical approach, widely targeted metabolomics, combines the “breadth” of untargeted metabolomics with the “accuracy” of targeted metabolomics. This method not only achieves higher throughput than untargeted metabolomics but also enables more precise identification and quantification of metabolites. In studies of drug components and drug metabolism, widely-targeted metabolomics has been successfully employed for large-scale metabolite profiling and comparative metabolomics across multiple species [21–24]. A metabolomics-based investigation of naringin and phlorizin in DSS-induced ulcerative colitis may help elucidate their therapeutic mechanisms.

To investigate the therapeutic effects and underlying mechanisms of naringin and phloridzin in DSS-induced UC, we applied an integrated widely targeted and targeted metabolomics approach to characterize metabolic alterations and identify differential metabolites in the present work. By comparing the therapeutic effects of naringin and phloridzin in a DSS-induced UC model, we reveal their distinct metabolic mechanisms. We further investigated the tryptophan metabolic pathway, which is closely associated with gut microbiota, in response to naringin and phloridzin treatment. This study underscores the pivotal role of tryptophan metabolism and microbiota-derived metabolites in maintaining intestinal health. The findings provide valuable insights into the metabolic mechanisms underlying the therapeutic effects of these flavonoids, offering a theoretical foundation for the development of novel UC treatment strategies based on natural compounds.

## Meterials and methods

### Material and chemicals

Methanol (CH_3_OH) (GR, ≥ 99%), acetonitrile (ACN) (GR, ≥ 99%) were purchased from Merk (German). Formylic acid (HCOOH) (GR, ≥ 98%), naringin (98.75%), phloridzin (98.55%) and dextran sulfate sodium (DSS) (AR) were purchased from Aladdin (Shanghai, China). L-2-phenylalanine (AR) was purchased from Brylwilliam (China). Sodium chloride injection (0.9%) was purchased from Wuhan Binhu Shuanghe Pharmaceutical Company. acetate, propionate, and butyrate, isobutyrate, n-valerate, isovalerate, n-caproate, and the internal standard 2,2-dimethylbutyrate were gas chromatographic standards purchased from Aladdin.

### Animals and design

**Fig. 1:**
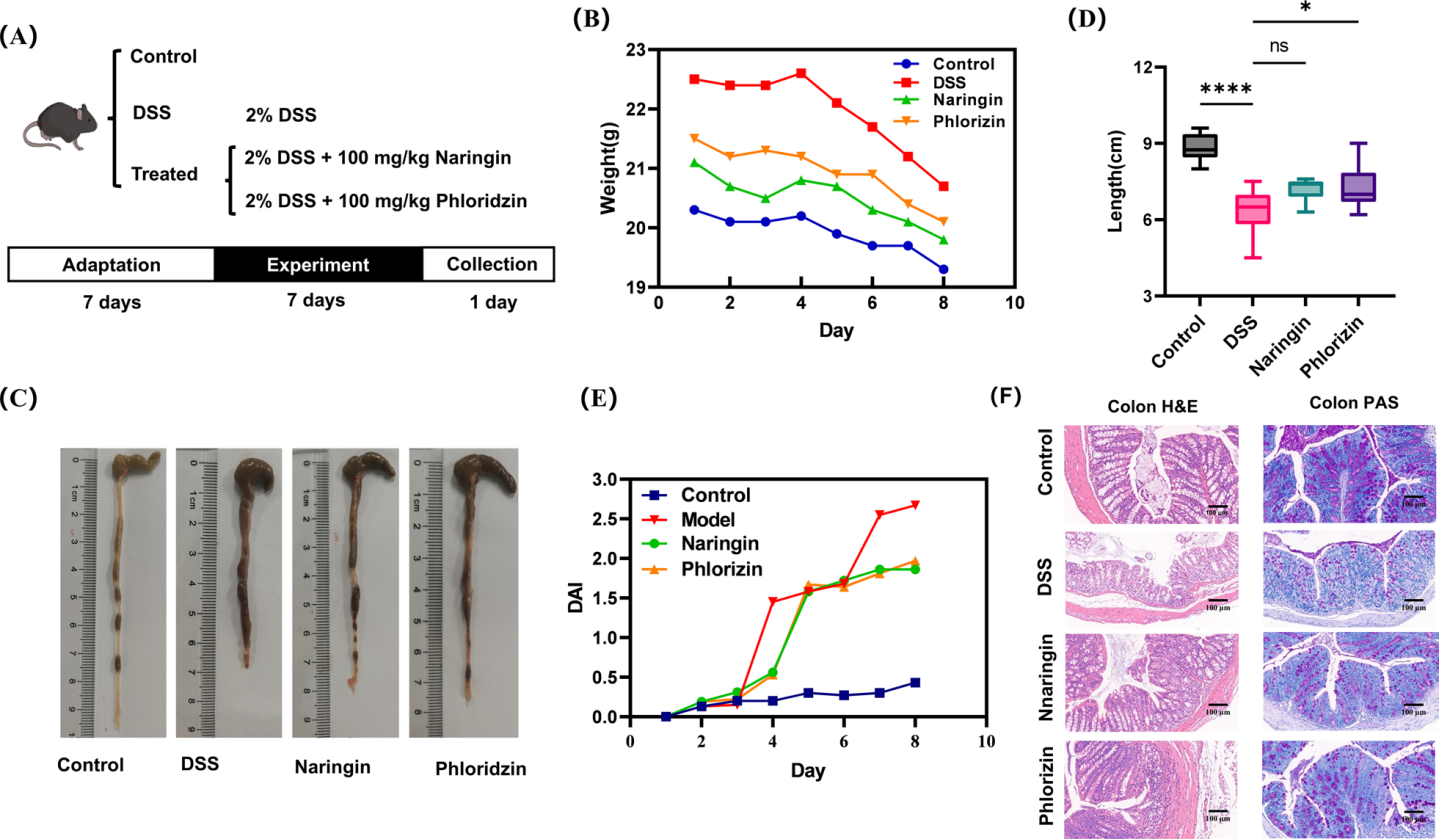
Experimental design of the DSS-induced UC mouse model and effects of naringin and phloridzin on UC mice. **A** Experimental design. **B** Body weight change measurement. **C** Colon length measurement(*, *p* < 0.05; ****, *p* < 0.0001, compared with the control group). **D** Macroscopic morphology of the colon. **E** DAI index. **F** Histological evaluation (H&E staining 100 ×)

A total of 54 male C57BL/6 mice (6–8 weeks old; 21–23 g) were purchased from Hunan SJA Laboratory Animal Co., Ltd. (Changsha, China), acclimatized for one week, and then randomly assigned to four groups: a Control group (n = 10), a DSS group (n = 20), a Naringin group (n = 12), and a Phloridzin group (n = 12). As outlined in the experimental design (Fig. 1A), ulcerative colitis was induced over seven days in the DSS, Naringin group and Phloridzin group by administering 2% (w/v) DSS in the drinking water, which was replaced every 48 h. Throughout this induction period, mice in the Naringin and Phloridzin groups received daily oral gavages of naringin (100 mg/kg) and phloridzin (100 mg/kg), respectively, while the Control group and DSS group received an equivalent volume of saline. On the morning of day 8, faecal samples were collected and stored at -80°C, after which the mice were dissected and colon tissues were harvested for subsequent analysis. All animal experiments were approved by the Committee on Care and Use of Laboratory Animals of South Central Minzu University, China (Approval No. 2022-scuec-38).

### Histopathological assessment

Harvested colon tissues were fixed in 10% neutral-buffered formalin, processed through a graded ethanol series for dehydration, and embedded in paraffin. The paraffin blocks were then sectioned and stained with haematoxylin and eosin (H&E) for histological evaluation.

### Histological immunochemistry and inflammatory factor assay

Quantitative detection of TNF-α, IL-17 and IL-22 levels in mucosal tissue and serum using commercial ELISA kits. Tissue samples were rinsed with PBS and homogenized in tissue protein extraction reagent. The homogenates were repeatedly shaken and pipetted, followed by centrifugation to collect the supernatant. Protein concentrations were determined using a BCA Protein Assay Kit. After sample preparation and antibody incubation, optical density values of the target protein bands were analysed with AlphaEaseFC software.

### Non-targeted metabolomics analysis

Briefly, the cecum content (40 mg) was extracted with the internal standard extract (containing 70% methanol). The supernatant was centrifuged three times at 12,000 rpm for 10 min, under freezing conditions and filtered through 0.22 μm polyvinylidene difluoride (PVDF) membrane for further analysis.

Non-targeted metabolomics employs ultra-performance liquid chromatography (UPLC) (ExionLC AD, https://sciex.com.cn/) and quadrupole-time-of-flight mass spectrometry (TripleTOF 6600, AB SCIEX). Column: Waters ACQUITY UPLC HSS T3 C18 1.8 µm, 2.1 mm × 100 mm. Flow rate: 0.35 ml/min; Column temperature: 40 °C; Injection volume: 5 µl. Positive: Mobile phase A was 0.1% formic acid in water and mobile phase B was 0.1% formic acid in acetonitrile. Negative: Mobile phase A was ultrapure water and mobile phase B was acetonitrile; Gradient conditions: 0–11 min, 5–90% B; 11–12 min, 90% B; 12–12.1 min, 90–5% B; 12.2–14 min, 5% B. Specific mass spectrometry conditions are detailed in the supporting information. Specific mass spectrometry conditions are detailed in the supporting information (Table S1).

### Widely targeted metabolomics analysis

The widely targeted metabolomics assay was performed by Metware (Wuhan, China). Widely-targeted metabolomics employs ultra-performance liquid chromatography (UPLC) (ExionLC AD, https://sciex.com.cn/) and tandem mass spectrometry (MS/MS) (QTRAP®, https://sciex.com/). Metabolite quantification was completed by analysis in MRM mode based on Q-Trap triple quadrupole mass spectrometry. Column: Waters ACQUITY UPLC HSS T3 C18 1.8 µm, 2.1 mm × 100 mm. Flow rate: 0.35 ml/min; Column temperature: 40 °C; Injection volume: 2 µl; Positive: Mobile phase A was 0.1% formic acid in water and mobile phase B was 0.1% formic acid in acetonitrile. Negative: Mobile phase A was ultrapure water and mobile phase B was acetonitrile; Gradient conditions: 0–11 min, 5%-90% B; 11–12 min, 90% B; 12–12.1 min, 90%-5% B; 12.2–14 min, 5% B. Mass spectrometry conditions same as in Sect. 2.5.

### Data analysis

Mass spectrometry data using public databases (including Metlin, HMDB, KEGG databases) and MetDNA, to obtain the multi-ion pair information and retention time of the identified metabolites. The most plausible batch of compounds was expanded for data analysis of the subsequent broad-target mass spectrometry assay. Metabolite quantification was completed by analysis in multiple reaction monitoring (MRM) mode based on Q-Trap triple quadrupole mass spectrometry. Data acquisition was performed using Analyst 1.6.3, and integration (quantification) was performed using MultiQuant 3.0.3 after down-conversion. Normalized data were subjected to Principal Component Analysis (PCA) and Orthogonal Partial Least Squares Discriminant Analysis (OPLS-DA) in R. Differential metabolites were screened based on the variable importance projection (VIP) of the OPLS-DA model in the multivariate statistical analysis, combined with the *P*-value and difference multiplicity value (FC) of the t-test of the univariate analysis, and the following criteria were used for the screening: VIP ≥ 1, FC ≥ 2 or FC ≤ 0.5, *P*-value ≤ 0.05. Pathway enrichment analysis was performed using the KEGG database. The diagnostic accuracy of potential biomarkers was assessed using subject working curves (ROC curves), and the relationship between DAI and potential biomarkers was analysed by linear regression.

### Targeted metabolomics analysis of tryptophan

20 mg of faecal sample mixed with 400 μL of internal standard extraction solution (70% methanol in water) and vortexed for 6 min, followed by sonication for 10 min. Next, the sample was vortexed again for 3 min and incubated at -20 °C for 60 min. After centrifuged at 12,000 rpm for 10 min, 200 μL of supernatant was injected into the analytical instrument for subsequent measurement and analysis.

The LC-QqQ-MS was used for the targeted detection of tryptophan pathway metabolites with an injection volume of 1 μL, column temperature of 40 °C, mobile phase A was 0.1% formic acid in water, mobile phase B was 0.1% formic acid in acetonitrile at a flow rate of 0.4 mL/min, and the chromatographic column was a Waters ACQUITY UPLC C18 + (2.1 × 50 mm, 1.7 µm). Gradient conditions: 0–2 min, 3–50% B; 2–2.5 min, 50–60% B; 2.5–3 min, 60–97% B; 3–3.5 min, 97–3% B; 3.5–5 min, 3% B. Detection was performed by negative multiple reaction monitoring (MRM). The nitrogen dry gas temperature was 300 °C and the nitrogen sheath gas temperature was 250 °C. The nitrogen dry gas flow rate was 5 L/min and the nitrogen sheath gas flow rate was 11 L/min. The capillary voltage was 3500 V, the nebulizer gas pressure was 45 psi, and the nozzle voltage was 500 V. The nozzle voltage was 500 V. The nozzles were designed to be used in a variety of applications. Specific MRM information is shown in Table S5.

### Targeted metabolomics analysis of SCFA

A total of 25 mg samples were mixed with 10 μL of internal standard (1 mM, 2,2-dimethylbutyric acid), 400 μL of 1 M HCl and then add 400 μL of EE after three freeze–thaw cycles. 200 μL of centrifugal separation supernatant (4 °C, 12,000 rpm, 10 min) was injected into the sample to be measured and analysed.

The 6890N-5973N Gas Chromatograph-Mass Spectrometer and a HP-INNOWAX (60 × 0.250 × 0.25 μm) gas chromatographic column was used, with helium as the carrier gas at a flow rate of 0.8 mL/min, and the inlet temperature was 250 °C, the ion source temperature was 230 °C, the capillary temperature was 250 °C, and the total injection time was 29 min: the temperature was 100 °C for 2 min; the temperature was increased to 120 °C and kept for 3 min, the temperature increase rate was 10 °C/min; the temperature was increased to 150 °C and kept for 6 min, the temperature increase rate was 510 °C/min; the temperature was increased to 250 °C and kept for 6 min, the temperature increase rate was 25 °C/min. Quantification was performed based on the relative peak area using acetate and 2, 2-dimethylbutyric acid as the internal standard.

## Results

### Naringin and phloridzin alleviated DSS-induced UC

After one week of treatment, body weight declined in all groups, including the Phloridzin group, Naringin group, and DSS group (Fig. 1B). Notably, weight loss was more pronounced in the DSS group compared with the Naringin and Phloridzin groups.

The severity of colitis was further assessed using the Disease Activity Index (DAI). Compared with the control group, DAI scores increased significantly with prolonged DSS exposure, and colon length was markedly reduced (Fig. 1C–E). Both naringin and phloridzin treatment alleviated these effects, partially restoring colon length and reducing DAI scores, although neither treatment fully normalized these parameters relative to the control group. It is noteworthy that Phloridzin demonstrates significant efficacy in restoring colon length (*p* < 0.05).

### Naringin and phloridzin repaired colonic tissue damage of UC

Further analysis of intestinal barrier damage was conducted using haematoxylin and eosin (H&E) staining and periodic acid–Schiff (PAS) staining. As depicted in Fig. 1E, DSS-treated mice displayed marked pathological alterations, including shortened intestinal mucosal epithelial villi, villous detachment, crypt deepening, mucosal edema, cellular infiltration, and decreased carbohydrate content. These findings indicate that DSS induction severely disrupted the normal structural integrity and function of colon cells in mice. Importantly, treatment with naringin and phloridzin demonstrated a pronounced ameliorative effect, significantly alleviating these pathological alterations. These results suggest that naringin and phloridzin play a protective role in restoring intestinal barrier function in UC mice.

### Naringin and phloridzin alleviated the immune response in the colon of UC

**Fig. 2:**
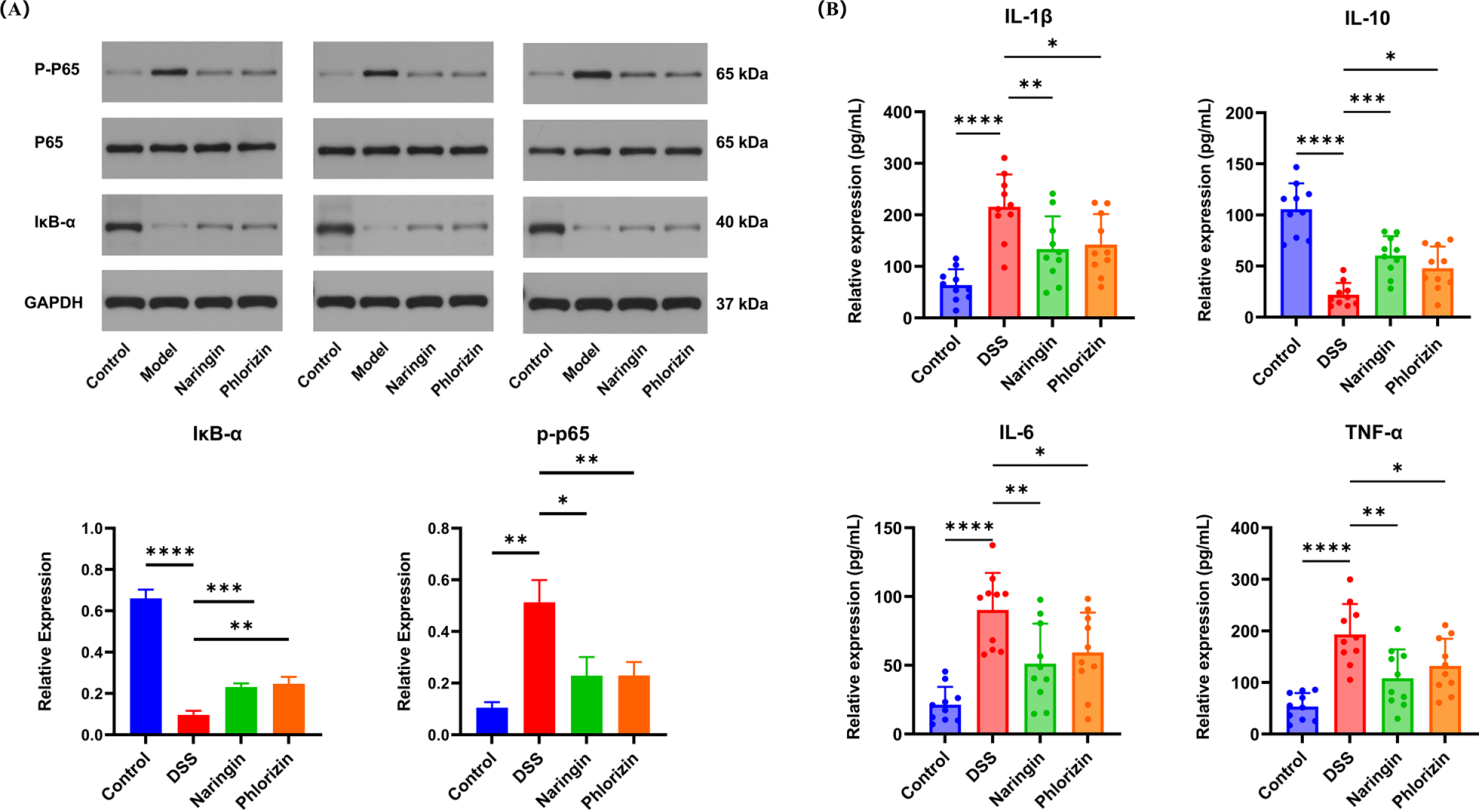
The immune response and inflammatory factors examination of the colons of UC mices treated with naringin and phloridzin. **A** Naringin inhibited the expression of IL-1β, TNF-α, IL-10 and IL-6 (n = 10). **B** Western blot for NF-κB signalling pathway (n = 3). *, *p* < 0.05; **, *p* < 0.01; ***, *p* < 0.001; ****, *p* < 0.0001, compared with the control group

Oxidative stress is a critical driver of colitis progression, and the Nuclear Factor Kappa-light-chain-enhancer of Activated B cells (NF-κB) Pathway plays a pivotal role in regulating inflammation. To further elucidate the underlying mechanisms, we detected key proteins of the NF-κB pathway in colonic tissues, including Phosphorylated Nuclear Factor—kappa B p65 (P-p65), Nuclear Factor—kappa B p65 Subunit (*p*-65), Inhibitor of κB alpha (IκB-α) and Glyceraldehyde-3-Phosphate Dehydrogenase (GAPDH). As shown in Fig. 2B and Fig. S1, DSS-induced UC mice exhibited significantly elevated levels of P-p65 and reduced levels of IκB-α compared to Control. Administration of naringin and phloridzin reversed these alterations, restoring P-p65 and IκB-α levels a great extent. Interestingly, both compounds demonstrated comparable efficacy in modulating the NF-κB pathway, highlighting their potential as therapeutic agents for mitigating inflammation and oxidative stress in UC.

By measuring the levels of inflammatory mediators downstream of the NF-κB pathway, the specific immunomodulatory effects of naringin and phloridzin in UC mice were investigated. As illustrated in Fig. 2A, DSS led mice to a significant increase in pro-inflammatory cytokines, including Tumour Necrosis Factor-α (TNF-α), Interleukin-1 β (IL-1β), and Interleukin-6 (IL-6), alongside a marked reduction in the anti-inflammatory cytokine Interleukin-10 (IL-10). Notably, both naringin and phloridzin treatments effectively counteracted these changes, significantly suppressing the upregulation of TNF-α, IL-1β, and IL-6 while mitigating the decline in IL-10 levels. From a numerical perspective, there was no significant difference between Naringin group and phloridzin group.

### Naringin and phloridzin regulated metabolism profiles in DSS-induced UC mice

**Fig. 3:**
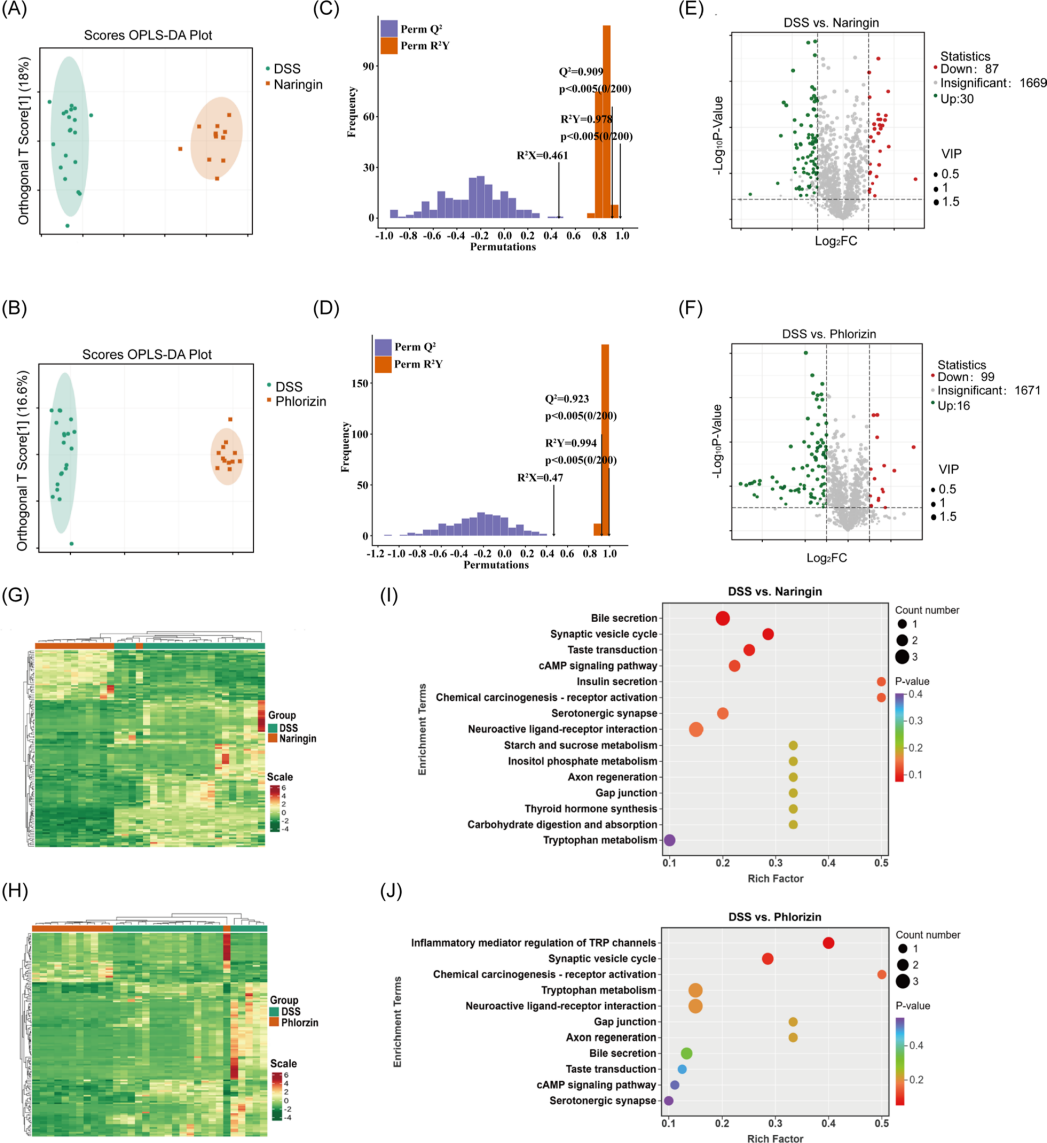
Multivariate analysis and pathway enrichment of metabolomic profiles in DSS-induced UC mice treated with naringin or phloridzin. The OPLS-DA of **A** DSS group vs Naringin group, **B** DSS group vs Phloridzin group (VIP > 1). Permutation Test of OPLS-DA of **C** DSS group vs Naringin group, **D** DSS group vs Phloridzin group. Values closer to 1 for R^2^X, R^2^Y, and Q^2^ indicate a better fit and higher predictive power. Volcano Plot of **E** DSS group vs Naringin group, **F** DSS group vs Phloridzin group. Red dots indicate a downward adjustment, green dots indicate an upward adjustment, and grey dots indicate no change. Hierarchical clustering heatmap of **G** DSS group vs. Naringin group, **H** DSS group vs. Phloridzin group. Colour indicates differential expression of metabolites (red: high expression; green: low expression). KEGG pathway enrichment analysis of **I** DSS group vs. Naringin group, **J** DSS group vs. Phloridzin group. Bubble size reflects the number of differential metabolites in each pathway; bubble colour represents − lg(*p*-value), with deeper colours indicating higher significance

A widely targeted metabolomics utilizing LC/MS was used to comprehensively analyse the metabolic profiles of faecal samples from mice across four distinct groups: Control, DSS, Naringin, and Phloridzin. The screening criteria of differential metabolites were set as a *p*-value < 0.05 in the t-test, with a Fold Change > 2 or 0.5. Supervised OPLS-DA models (VIP > 1) were constructed to further evaluate differences between the control and treatment group. Partial least squares discriminant analysis (OPLS-DA) was used to identify key differential metabolites between groups by eliminating irrelevant variables. In the model, R^2^X and R^2^Y represent the explained variance of the X and Y matrices, respectively, while Q^2^ indicates the model's predictive ability. Values closer to 1 for R^2^X, R^2^Y, and Q^2^ indicate a better fit and higher predictive power. OPLS-DA models showed clear separation between Naringin group, Phloridzin group and DSS group with cross validation parameters of good quality (Fig. 3A–D and Fig. S2A-B). In the DSS group and Control group, 583 differential metabolites were identified, comprising 178 upregulated and 405 downregulated metabolites (Fig. S2C). Compared with the DSS group, the Naringin group identified 117 differential metabolites, including 30 upregulated and 87 downregulated metabolites (Fig. 3E); the Phloridzin group identified 115 differential metabolites, with 16 upregulated and 99 downregulated metabolites (Fig. 3F).

The heatmap based on cluster analysis revealed significant metabolic differences between the groups (Fig. 3G–H and Fig. S2C). It suggested that naringin, and phloridzin induced substantial alterations in the metabolic pathways of UC mice. Although naringin and phloridzin both belong to the flavonoid compound family, their effects on the metabolism of UC mice are not entirely identical (Fig. S2D). Analysis of these differential metabolites into the KEGG pathway showed that DSS (Fig. S5) caused disruption of multiple metabolic pathways such as amino acid biosynthesis, ATP-binding cassette transporter (ABC transporter), arginine biosynthesis, pyrimidine metabolism, purine metabolism and central carbon metabolism in cancer. The metabolic pathways associated with naringin treatment (Fig. 3I) are tryptophan metabolism, purine metabolism, bile secretion, thyroxine synthesis, synaptic messaging pathways et al. And the metabolic pathways associated with phloridzin treatment (Fig. 3J) include tryptophan metabolism, bile secretion, neuroactive ligand receptor activation, and cyclic adenosine monophosphate signalling pathway et al. Notably, both naringin and phloridzin treatments had significant regulatory effects on the tryptophan metabolic pathway.

### Naringin and phloridzin improved UC by regulating tryptophan metabolism

**Fig. 4:**
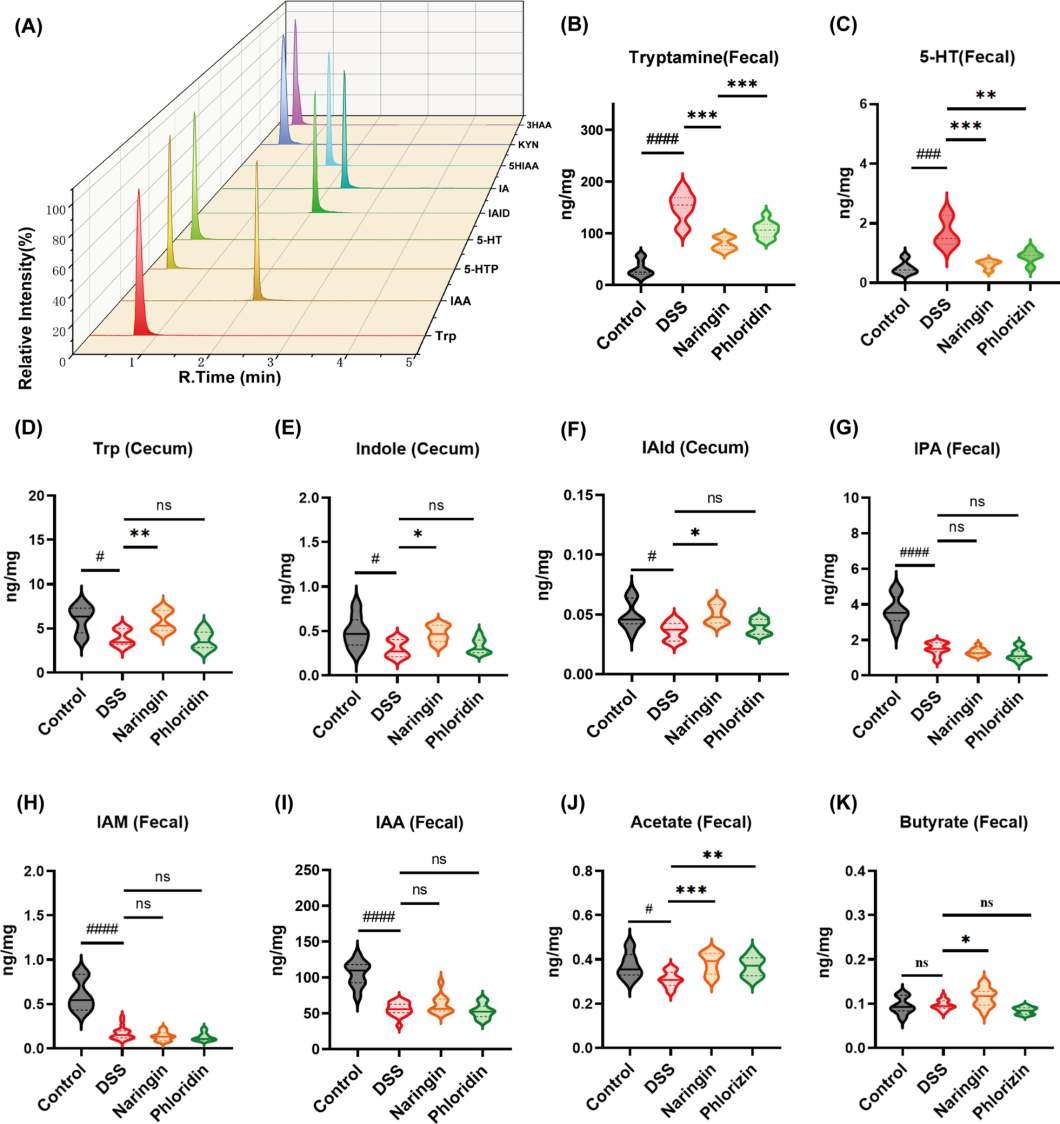
Quantitative analysis of the regulatory effects of naringin and phloridzin on tryptophan (Trp) metabolism and its associated metabolites. **A** MRM chromatograms of targeted metabolomics on tryptophan metabolites. Changes in tryptophan metabolism of **B** Tryptamine, **C** IAA, **D** IAM, **E** 5-HT, **F** IAld, **G** IPA, **H** Trp, **I** Indole. Naringin and phloridzin increase the **J** Acetate. **K** Butyrate in SCFAs. (# or *, *p* < 0.05; ## or **, *p* < 0.01; *** or ###, *p* < 0.001; ####, *p* < 0.0001; ns, not significant)

To elucidate potential similarities and distinctions in naringin and phloridzin regulatory mechanisms, we performed targeted quantification of Tryptophan and its downstream metabolites in murine faecal samples, with the resulting MRM chromatograms presented in Fig. 4A. As illustrated in Fig. 4B–I, Tryptamine and 5-HT emerged as differential metabolites both in Naringin group and phloridzin group relative to DSS group. Furthermore, DSS administration induced marked dysregulation of the indole pathway in tryptophan metabolism, characterized by significant reductions in Tryptophan (Trp), Indole, indole-3-aldehyde (IAld), indole-3-propionic acid (IPA), indole-3-acetamide (IAM), indole acrylic acid (IAA) and increase in Tryptamine and 5-HT. Naringin intervention partially restored intestinal metabolic homeostasis, with upregulated levels of indole, Trp, IAld and down in Tryptamine and 5-HT observed in Naringin group. In contrast, phloridzin exert a pronounced regulatory effect solely upon the levels of Tryptamine, and 5-HT, suggesting divergent mechanisms of action between the two flavonoids.

### Naringin and phloridzin increased the production of SCFAs

The metabolic degradation of dietary fibres by gut microbiota generates a range of metabolic byproducts, including organic acids, gases, and substantial amounts of short-chain fatty acids (SCFAs). Accumulating evidence from numerous studies has demonstrated the therapeutic potential of SCFAs in alleviating UC. The quantitative analysis of acetate, propionate, and butyrate, isobutyrate, n-valerate, isovalerate, n-caproate in faecal samples collected from four experimental mouse groups.

As shown in Fig. 4 J and K, both the Naringin group and phloridzin group exhibited elevated acetate levels compared to DSS group, though these values remained below those observed in control group. While no statistically significant differences in propionate and butyrate content were detected between DSS group and control group, naringin demonstrated a marked increase in butyrate concentration relative to other experimental groups. Importantly, naringin and phloridzin effectively improved the DSS-induced reduction in acetate. Hence, these experimental observations indicate that naringin and phloridzin may ameliorate ulcerative colitis (UC) through modulation of SCFAs biosynthesis.

## Discussion

Our results demonstrate that oral administration of naringin and phloridzin effectively mitigated key UC phenotypes in mice. Both compounds attenuated weight loss, preserved colon length, lowered the DAI score, and reduced histological damage. These physiological improvements were accompanied by suppressed NF-κB pathway activity and reduced serum inflammatory factor levels. Collectively, these findings show that naringin and phloridzin possess significant therapeutic potential for UC management, and their overall outcomes were comparable. Through metabolomic analysis, we discovered that naringin and phloridzin exerted differing effects on the metabolism of UC mice. Further targeted analysis revealed that naringin upregulated the levels of Trp, Indole, IAld in tryptophan metabolism, alongside acetate and butyrate of SCAF. In contrast, apart from acetic acid, phloridzin showed any regulatory effect on these metabolites.

In this study, mice in the DSS group exhibited significant weight loss. Their colons were shortened, and DAI scores were increased, intestinal tissue damage was also observed. Metabolomic profiling analysis revealed significant disruption of the ABC transporter metabolic pathway in DSS-treated mice. ABC transporter proteins, which are crucial efflux transporters associated with the intestinal mucosa, play a pivotal role in the transport of nutrients and waste products, energy production, and cellular communication. Previous studies have suggested that altered expression of ABC transporter proteins may be implicated in the pathogenesis of IBD [25]. Furthermore, the analysis revealed significant disturbances in pyrimidine and purine metabolism in UC mice. Extensive research has established that dysregulated purine metabolism is closely associated with excessive inflammation in IBD [26–28]. These findings align with existing literature, confirming the successful establishment of the UC model.

The experimental findings demonstrate that naringin and phloridzin exert therapeutic effects in UC. Specifically, both compounds attenuate weight loss, prevent colonic shortening and intestinal tissue damage, and suppress hyperactivated inflammatory and immune responses. Compared with the model group, naringin- and phloridzin-treated mice exhibited significantly decreased expression of the pro-inflammatory cytokines TNF-α, IL-1β, and IL-6, along with markedly increased expression of the anti-inflammatory cytokine IL-10. These findings indicate effective restoration of inflammatory homeostasis. Concurrently, both treatments significantly reduced *p*-p65 expression within the NF-κB pathway while markedly increasing levels of the inhibitory protein IκB-α. Collectively, these results indicate that naringin and phloridzin exert anti-inflammatory effects in UC by suppressing aberrant NF-κB pathway activation. This mechanism suppresses the release of pro-inflammatory cytokines and enhances anti-inflammatory cytokine expression, thereby restoring inflammatory homeostasis. Inflammatory mediators are pivotal for maintaining organismal health; the balance between pro- and anti-inflammatory cytokines not only contributes to immune defence but also regulates tissue repair and the progression of pathological damage. Histological analysis using H&E and PAS staining revealed that naringin- and phloridzin-treated mice exhibited substantially reduced intestinal pathology. Mucosal structural integrity was largely restored. Compared with the DSS group, the number of goblet cells increased significantly in both treatment groups. The mucus layer was thickened, and its continuity was restored. These findings indicate effective protection of the intestinal barrier structure and mucus defence function. In summary, molecular and histological evidence collectively confirm that naringin and phloridzin modulate inflammatory balance by targeting and inhibiting the NF-κB pathway. These compounds reduce inflammatory damage to the intestinal barrier, promote its repair, and ultimately ameliorate the pathological features of DSS-induced colitis.

The present study demonstrates that both naringin and phloridzin modulate tryptophan metabolism, indicating a potential metabolic mechanism underlying their protective effects in UC. Widely targeted metabolomics analysis revealed significant reductions in tryptamine and serotonin (5-HT) following treatment, indicating a regulatory effect on the serotonin branch of tryptophan metabolism. Serotonin (5-HT) is synthesized from tryptophan via tryptophan hydroxylase (TPH), the rate-limiting enzyme that governs intestinal motility, secretion, and mucosal signaling. Emerging evidence suggests that dysregulated 5-HT signalling contributes to intestinal inflammation. Chan et al. reported that abnormally elevated 5-HT levels in mice induced lymphocyte activation and triggered excessive release of pro-inflammatory cytokines, a pattern closely resembling the pathological features of human inflammatory bowel disease (IBD) [29]. This inflammatory response pattern closely parallels the pathological characteristics observed in human inflammatory bowel disease. Previous studies further indicate that elevated 5-HT levels and increased TPH expression are associated with the progression of UC-related inflammation toward colorectal carcinoma [30]. Consistent with these findings, our data showed that naringin and phloridzin reduced 5-HT levels in DSS-induced UC mice. This reduction may be mediated through modulation of TPH activity or expression, thereby attenuating inflammation and improving intestinal function.

**Fig. 5:**
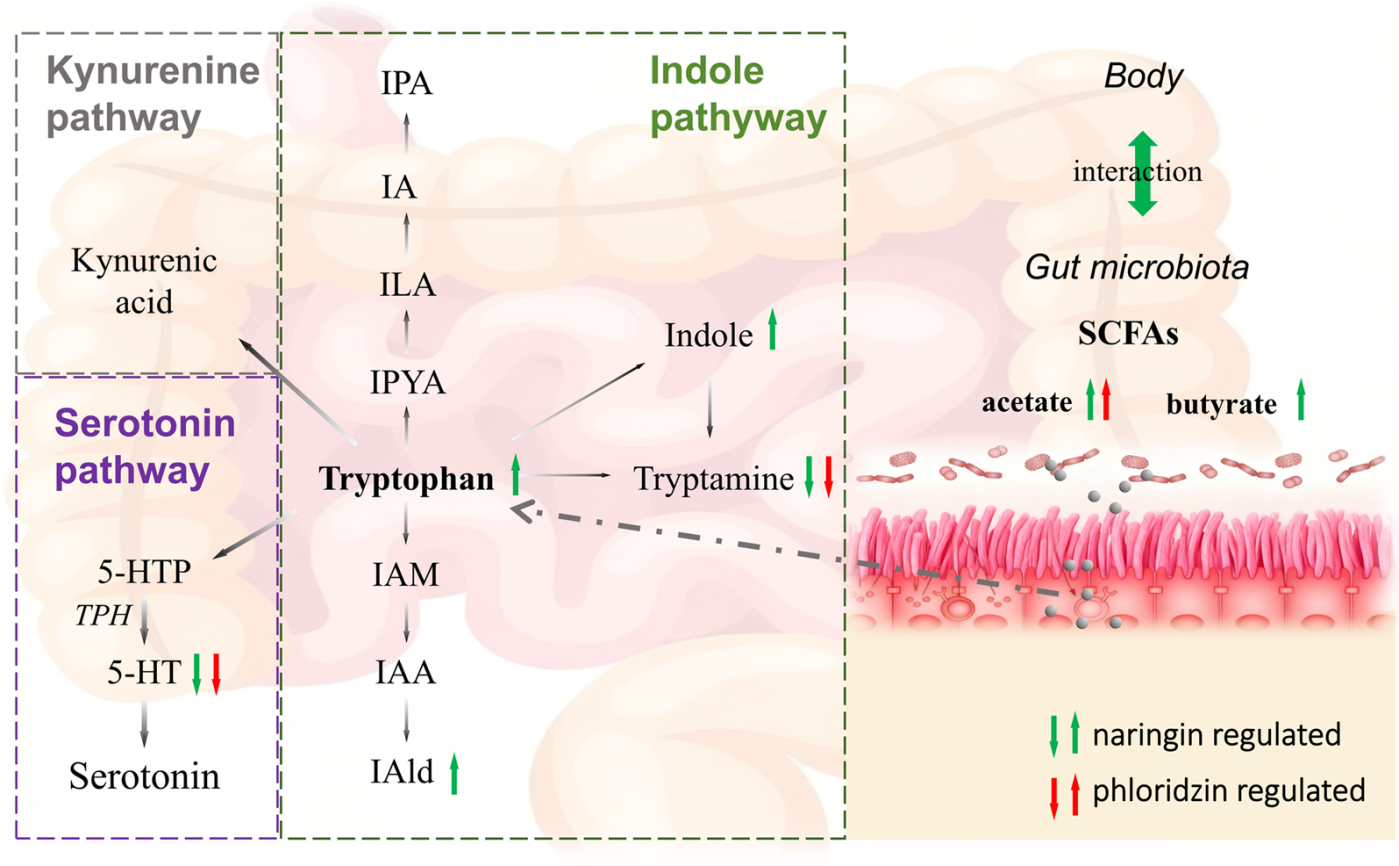
Schematic illustration of naringin and phloridzin-mediated regulation of tryptophan and SCFA metabolism in UC mice

Dietary tryptophan metabolism proceeds through three major pathways: the kynurenine pathway, the serotonin pathway, and the indole pathway (Fig. 5). Moreover, decreased tryptophan levels, coupled with increased conversion to tryptamine and 5-HT, disrupt the metabolic equilibrium of tryptophan. Previous studies have shown that tryptophan (Trp) levels are associated with inflammatory bowel disease [31, 32]. Targeted metabolomics further indicated that DSS disrupted the indole pathway of Trp metabolism, resulting in decreased levels of Trp, IPA, IAM, indole, IAld, and IAA. Naringin reversed these decreases, particularly restoring levels of indole, IAld, and Trp, whereas phloridzin showed no significant effect on these metabolites. Wang et al. reported that decreased levels of tryptophan-derived indole metabolites are associated with inflammation in intestinal mucosa and compromised intestinal barrier function [33]. Another study demonstrated that IAID administration reduced IL-6, IL-1β, and TNF-α protein levels in colonic tissues of colitis-induced mice and in LPS-stimulated macrophages [34]. Therefore, based on these findings, we propose that naringin may exert anti-inflammatory effects by restoring the levels of tryptophan-indole metabolites.

According to the observations on targeted metabolomics of SCFAs, phloridzin could significantly increase acetate, whereas naringin increased both acetate and butyrate. Sam et al. demonstrated that SCFAs, including acetate, propionate, and butyrate, alleviate inflammatory responses by reducing IL-1β, IL-6, and TNF-α levels [35].SCFAs also act as mediators in host–microbiota co-metabolism and can indirectly enhance tryptophan metabolism. Furthermore, intestinal SCFAs are primarily produced through the fermentation of dietary fibre by gut microbiota. Large quantities of unabsorbed and unmetabolized flavonoids entering the colon exert an indirect regulatory effect on the gut microbiota, thereby modulating its metabolic characteristics and functional activity. Therefore, the observed increases in SCFA levels suggest that naringin and phloridzin may beneficially modulate gut microbiota, a hypothesis that is scientifically plausible.

## Conclusion

In conclusion, this study demonstrates that naringin and phloridzin exert protective effects against DSS-induced ulcerative colitis by modulating tryptophan metabolism and suppressing inflammation through inhibition of the NF-κB signaling pathway. By integrating UPLC–MS/MS–based widely targeted metabolomics with targeted analyses of tryptophan and short-chain fatty acids, we systematically characterized the metabolic alterations associated with flavonoid intervention and elucidated their distinct regulatory mechanisms.

Both compounds reduced the levels of pro-inflammatory tryptophan metabolites, including serotonin and tryptamine, while promoting SCFA production, particularly acetate. Notably, naringin induced more extensive metabolic alterations than phloridzin, particularly in microbiota-associated tryptophan and SCFA pathways, characterized by elevated IALD, IAA, and butyrate levels, thereby promoting gut metabolic homeostasis.

Collectively, these findings highlight the pivotal role of microbiota-associated tryptophan metabolism in UC pathogenesis and recovery, and underscore the value of combined metabolomics strategies in uncovering mechanism-level insights. Dietary interventions based on flavonoid supplementation, along with prebiotic or probiotic approaches aimed at optimizing beneficial tryptophan metabolites, may therefore represent promising therapeutic strategies for ulcerative colitis.

Given the relatively short duration of naringin and phloridzin treatment in the present study, longer-term investigations are required to fully assess their therapeutic efficacy and elucidate their roles in regulating microbial tryptophan metabolism. Nevertheless, this study provides compelling evidence supporting the therapeutic potential of these natural compounds for the treatment of UC. Natural products such as naringin and phloridzin therefore hold substantial promise for the development of safer and more effective therapeutic agents, offering valuable alternative or complementary treatment options for patients with inflammatory bowel disease.

## Supplementary Information

Additional file 1.

## Data Availability

All data needed to evaluate the conclusions of this study are present in the paper.

## Abbreviations

DSS: Dextran sulphate sodium
TPH: Tyrosine phosphotransferase
TRP: Transient receptor potential
AhR: Aryl hydrocarbon receptor
cAMP: Cyclic adenosine monophosphate
Trp: Tryptophan
3HAA: 3-Hydroxykynurenine
3H-Kyn: 3-Hydroxynurenine
5-HIAA: 5-Hydroxyindole-3-acetic acid
5-HTP: 5-Hydroxytryptophan
5-HT: Serotonin
IA: Indole acrylic acid
IAA: Indole acetic acid
IAM: Indole-3-acetamide
IAAld: Indole-3-acetaldehyde
IAld: Indole-3-aldehyde
IPA: Indole-3-propionic acid
IPYA: Indole-3-pyruvate
ILA: Indole-3-lactic acid
