# Supplementary material for: Elucidating therapeutic mechanisms of naringin and phloridzin on ulcerative colitis mice by metabolomics-based comparative analysis
Source: Nat Prod Bioprospect. 2026 Jun 1;16(1):68. doi: 10.1007/s13659-026-00620-4 (PMC13226742; doi:10.1007/s13659-026-00620-4)
Supplement: Supplementary file 1 — Additional file 1. [file 13659_2026_620_MOESM1_ESM.docx]

**Supporting Information**

**(13 page, 4 figures, 4 tables)**

**Elucidating Therapeutic Mechanisms of Naringin and Phloridzin on Ulcerative Colitis Mice by Metabolomics-Based Comparative Analysis**

**Contents**

**Supplementary Figures**

**Figure S1.** Raw data from Western blot analysis of the NF-κB signaling pathway.

**Figure S2.** The OPLS-DA and volcano plot of DSS group vs. Naringin group and Naringin group vs. Phloridzin group.

**Figure S3** Heatmap of DSS group vs. Naringin group and Naringin group vs. Phloridzin group.

**Figure S4** Bubble plot of Control group vs. DSS group.

**Supplementary Tables**

**Table S1** KEGG pathway enrichment annotation of differential metabolites.

**Table S2** Top differential metabolites of DSS group VS. Naringin group.

**Table S3** Top differential metabolites of Phloridzin group VS. DSS group.

**Table S4** MRM information of TRP metabolites.

**
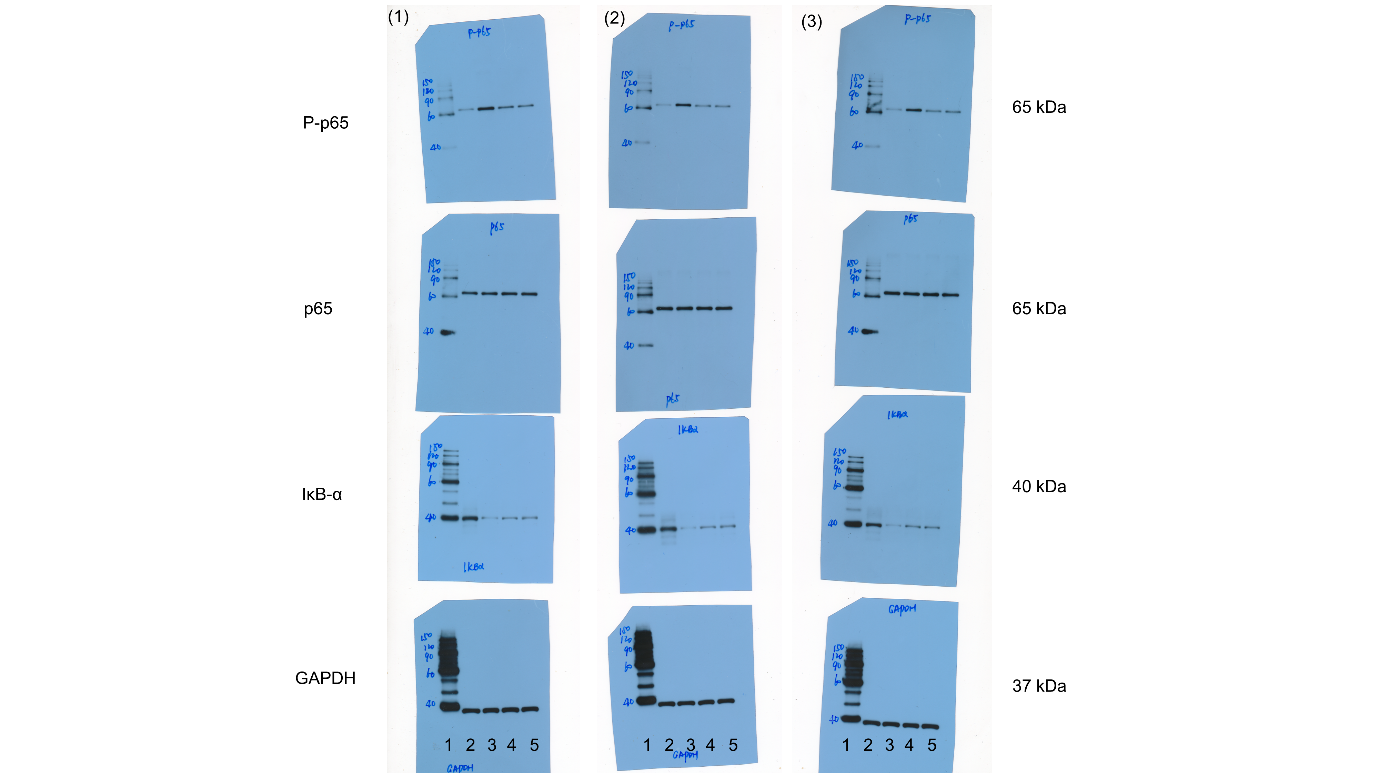
**

**Figure. S1** Raw data from Western blot analysis of the NF-κB signaling pathway (n=3). Lane 1 is the reference; Lane 2 is the control group；Lane 3 is the DSS group; Lane 4 is the naringin group；Lane 5 is the phloridzin group.

**
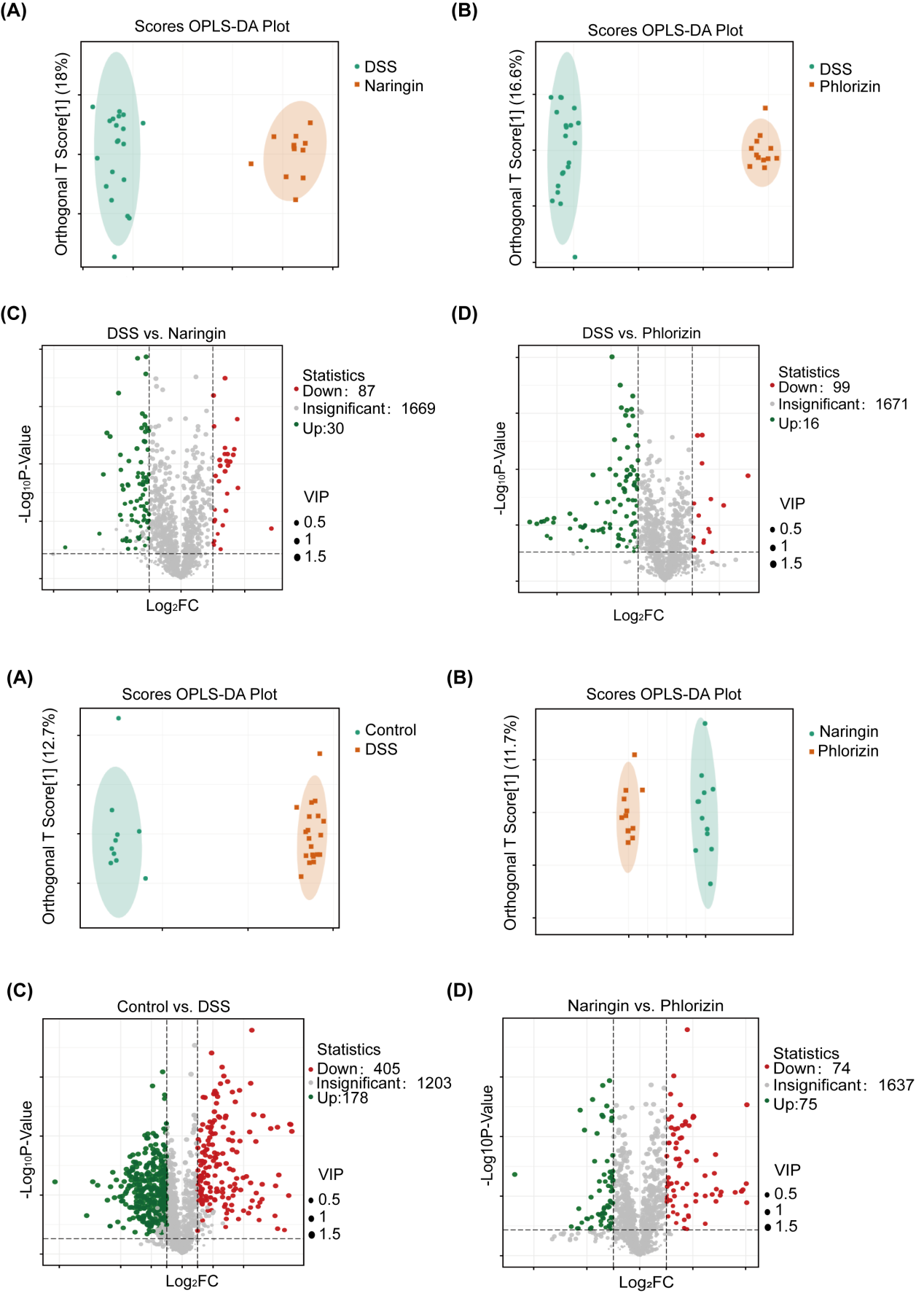
**

**Figure. S2** The OPLS-DA of (A) Control group vs DSS group, (B) Naringin group vs Phloridzin group (VIP>1.) and volcano plot of (C) Control group vs DSS group, (D) Naringin group vs Phloridzin group. Red dots indicate a downward adjustment, green dots indicate an upward adjustment, and gray dots indicate no change.


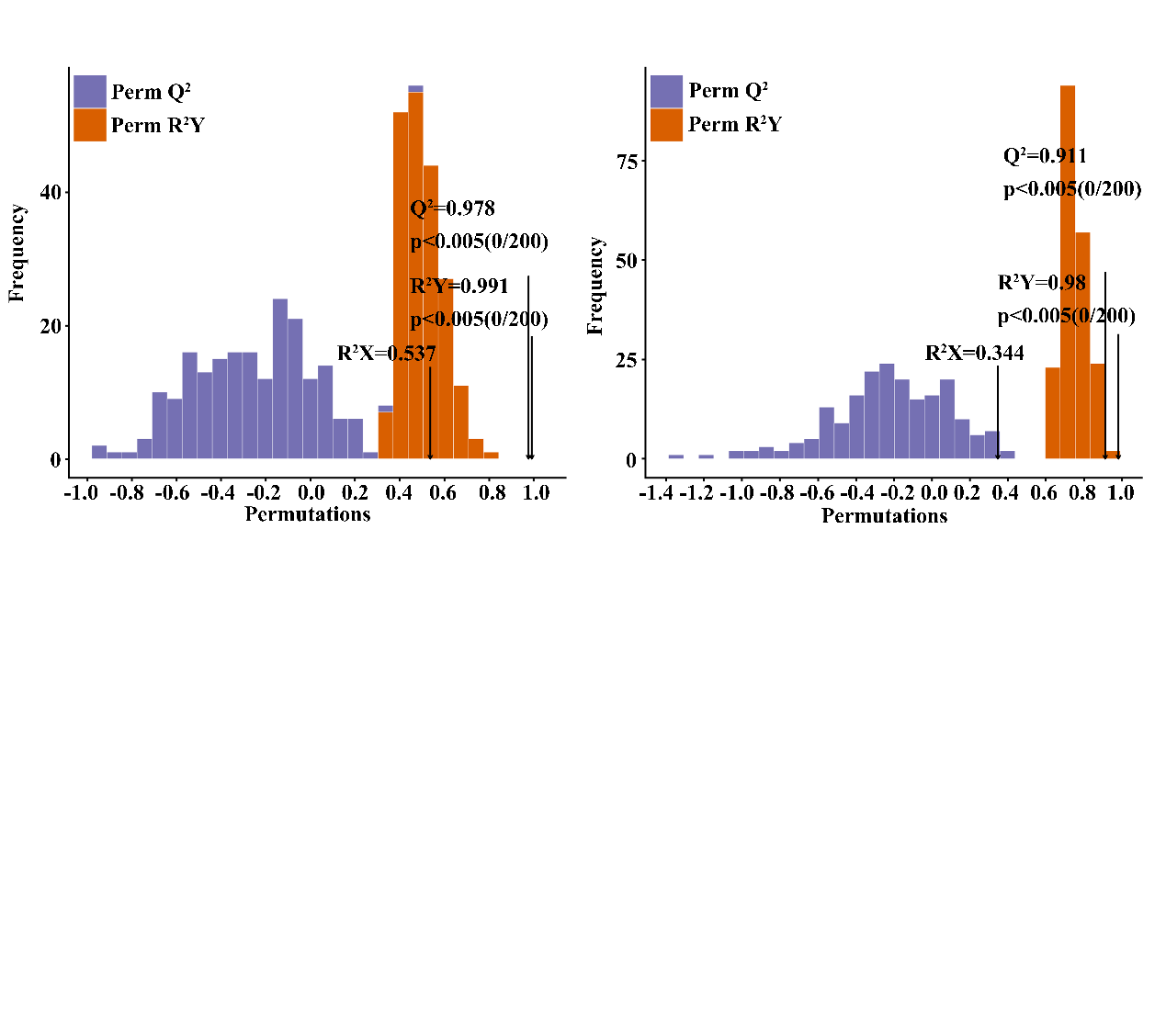


**Figure. S3** OPLS-DA validation diagram of (A) DSS group vs Naringin group, (B) Naringin group vs Phloridzin group.

**
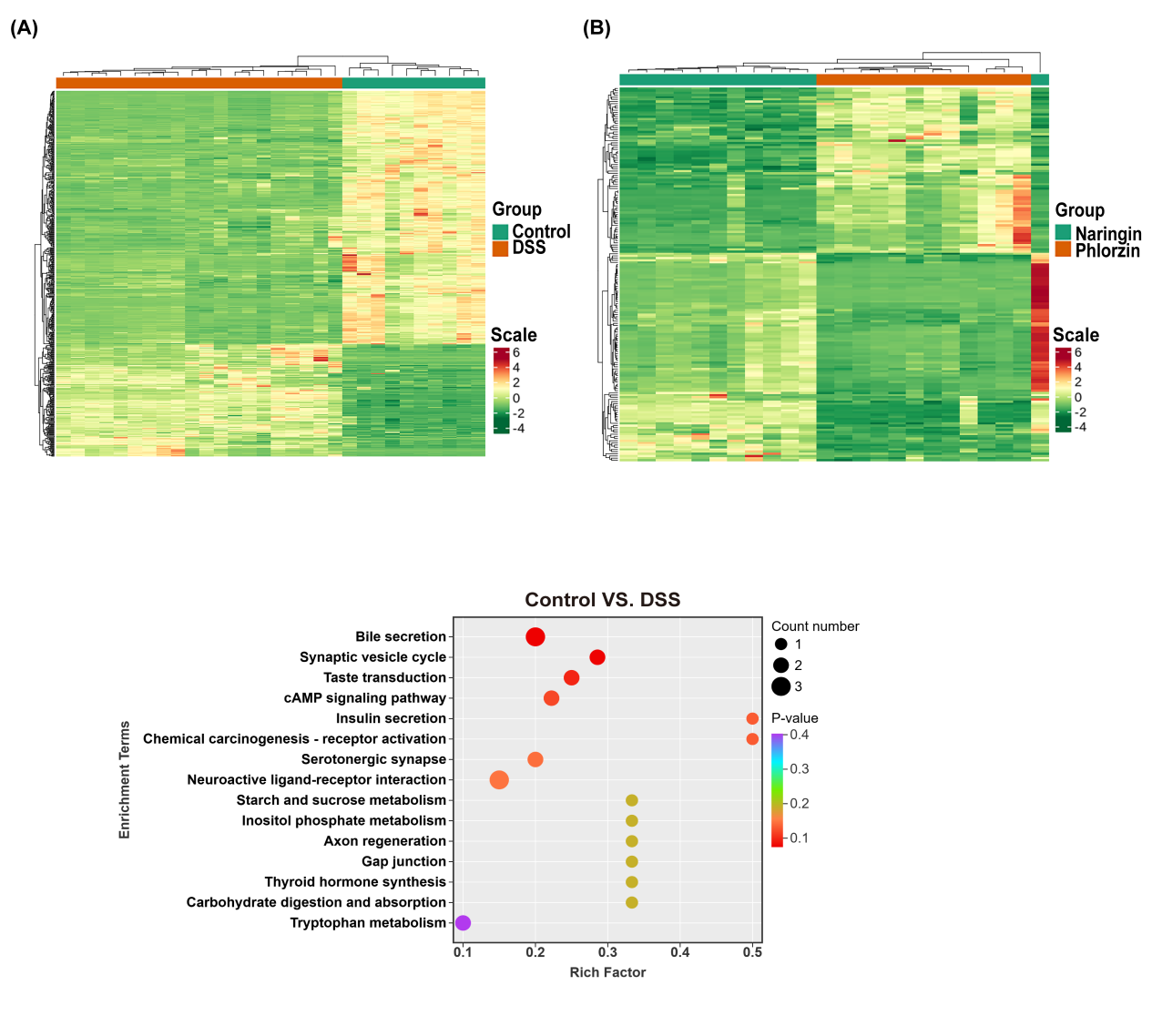
**

**Figure. S4** Heatmap of (A) Control group vs. DSS group, (B) Naringin group vs. Phloridzin group. Colour indicates differential expression of metabolites (red: high expression; green: low expression)

**
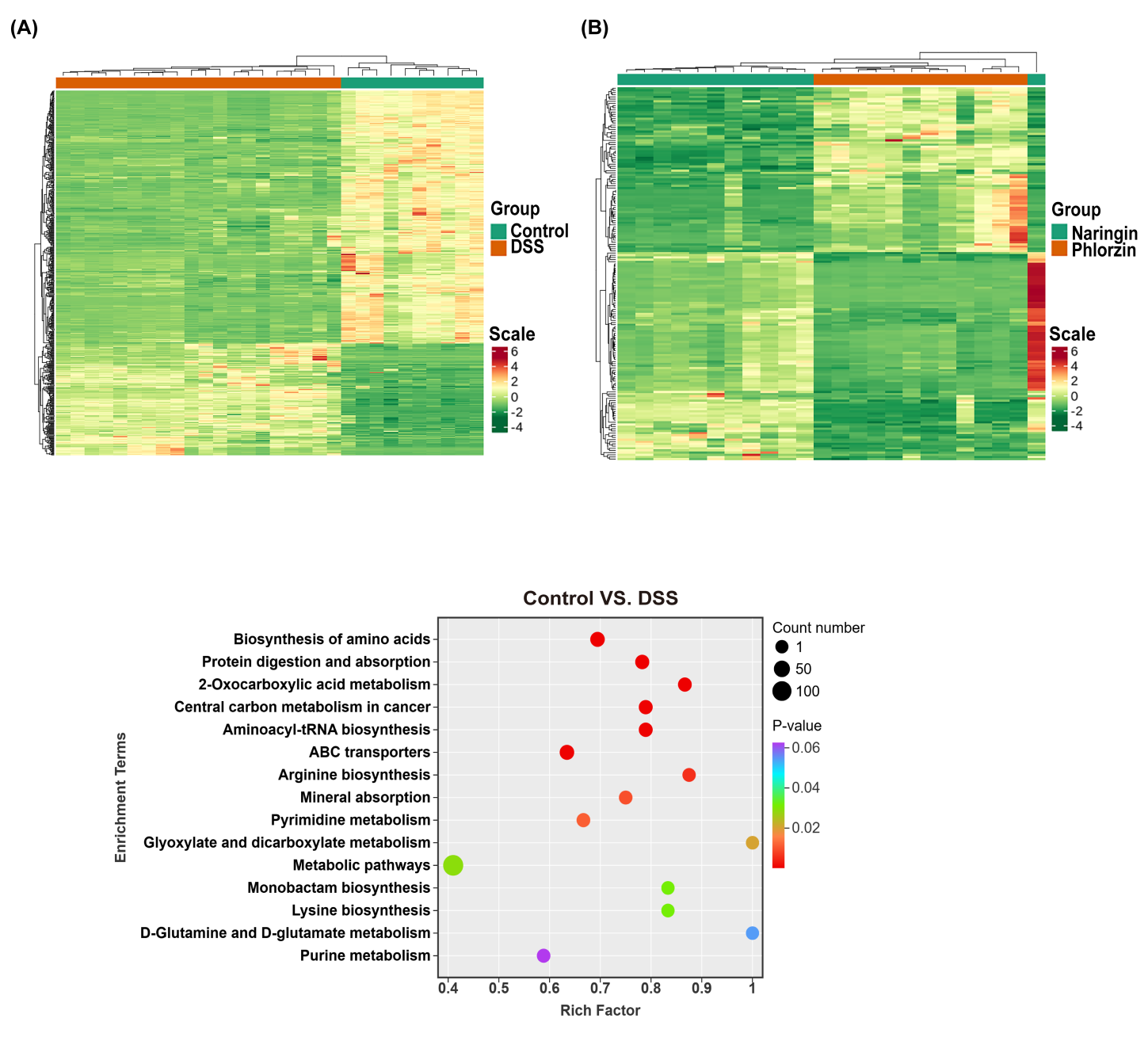
**

**Figure. S5** Bubble plot of Control group vs. DSS group. Bubble size reflects the number of differential metabolites in each pathway; bubble color represents −lg(p-value), with deeper colors indicating higher significance.

Table1: Mobile phase gradient conditions in positive and negative ion modes

| 时间（min） | 流速（mL/min） | A (%) | B (%) |
| --- | --- | --- | --- |
| 0.0 | 0.35 | 95 | 5 |
| 10.0 | 0.35 | 10 | 90 |
| 11.0 | 0.35 | 10 | 90 |
| 11.1 | 0.35 | 95 | 5 |
| 14.0 | 0.35 | 95 | 5 |

Table2: Mass spectrometry conditions in positive and negative ion modes

| Parameter | ESI+ | ESI- |
| --- | --- | --- |
| Curtain Gas | 25 | 25 |
| IonSpray Voltage | 5500 | 4500 |
| Temperature | 500 | 500 |
| Ion Source Gas1 | 50 | 50 |
| Ion Source Gas2 | 50 | 50 |
| Declustering Potential | 80 | -80 |
| Collision Energy | 30 | -30 |
| Collision Energy Spread | 15 | 15 |

In the Qtrap, each ion pair is scanned and detected based on an optimised declustering potential and collision energy.

**Table S3** KEGG pathway enrichment annotation of differential metabolites

| **Group Name** | **Total**  **differential metabolites*** | **Down-regulated** | **Up-regulated** |
| --- | --- | --- | --- |
| Phlorizin_vs_Naringin | 172 | 96 | 76 |
| Control_vs_Naringin | 649 | 464 | 185 |
| Control_vs_Phlorizin | 677 | 484 | 193 |
| Model_vs_Naringin | 118 | 88 | 30 |
| Model_vs_Phlorizin | 132 | 100 | 32 |
| Control_vs_Model | 584 | 405 | 179 |

***** The screening criteria of differential metabolites were set as a p-value < 0.05 in the t-test, with a Fold Change > 2 or 0.5.

**Table 4**. Top differential metabolites of DSS VS Naringin

| **Compound*** | **Trend** | **KEGG_map** |
| --- | --- | --- |
| D-Glucose 6-Phosphate | Control＞model＜Naringin | Ko04911-Insulin secretion  Ko562-Inositol phosphate  Ko04918-Thyroid hormone synthesis  ko04973-Carbohydrate digestion and absorption  ko01100-Metabolic pathway |
| Serotonin  (5-HT) | Control＜model＞Naringin | ko04750-Inflammatory mediator  ko00380-Tyrptophan metabolism  ko04080-Neuroactive ligand-receptor interaction  ko04976-Bile acid |
| 2'-Deoxyadenosine-5'-Monophosphate | Control＜model＞Naringin | ko00230-Purine metabolism  ko01100-Metabolic pathway |
| Tryptamine | Control＜model＞Naringin | ko00380-Tyrptophan metabolism  ko01100-Metabolic pathway |
| PGJ2 | Control＜model＞Naringin | ko00590-Arachidonic acid metabolism  ko01100-Metabolic pathway  ko04726-Serotonergic synapse |
| 3-Methylcatechol | Control＞model＜Naringin | ko01100-Metabolic pathway |

***** The screening criteria of differential metabolites were set as a p-value < 0.05 in the t-test, with a Fold Change > 2 or 0.5.

**Table 5.** Top differential metabolites of DSS VS Phlorizin.

| **Compound*** | **Trend** | **KEGG_map** |
| --- | --- | --- |
| 9E,11E-Octadecadienoic acid | Control ＜model＞ Phlorizin | ko00591-Linoleic acid metabolism |
| (2R,3R)-3-Methylglutamyl-5-semialdehyde-N6-lysine | Control ＞model＜ Phlorizin | ko00300-Lysine biosynthesis  Ko0110-Metabolic pathway |
| Serotonin | Control ＜model＞ Phlorizin | Ko04750-Inflammatory mediator  Ko00380-Tyrptophan metabolism  Ko04080-Neuroactive ligand-receptor interaction  Ko04976-Bile acid |
| Tryptamine | Control ＜model＞ Phlorizin | Ko00380-Tyrptophan metabolism  Ko01100-Metabolic pathway |
| P-Toluenesulfonic acid | Control ＜model＞ Phlorizin | Ko01100-Metabolic pathway |

***** The screening criteria of differential metabolites were set as a p-value < 0.05 in the t-test, with a Fold Change > 2 or 0.5.

**Table 6** MRM information of Trp metabolites.

| **Compound Name*** | **precursor ion（m/z）** | **Product ion（m/z）** | **Q1 (V)** | **CE (V)** | **Q3 (V)** |
| --- | --- | --- | --- | --- | --- |
| Trpd5(IS) | 210 | 150 | -15 | -17 | -15 |
| Trp | 205 | 188 | -14 | -9 | -20 |
|  | 205 | 146 |  |  |  |
| TA | 161.1 | 144.1 | -15 | -16 | -30 |
|  | 161.1 | 115 | -30 | -13 | -30 |
| IAA | 176.1 | 130.1 | -12 | -39 | -12 |
| QA | 124 | 106 | -13 | -16 | -26 |
|  | 124 | 96 |  |  |  |
| IPA | 190 | 130 | -14 | -15 | -20 |
| 5-HIAA | 192.1 | 146 | -15 | -17 | -17 |
| IAM | 175 | 130 | -10 | -20 | -24 |
| TRO | 162 | 144 |  |  |  |
| 3HAA | 154 | 108 | -10 | -16 | -15 |
|  | 154 | 80 |  |  |  |
| 5-HTP | 221 | 204 | -14 | -16 | -13 |
|  | 221 | 162 |  |  |  |
| 5-HT | 177 | 160 | -13 | -15 | -15 |
|  | 177 | 132 |  |  |  |
| KYN | 209 | 146 | -18 | -22 | -19 |
|  | 209 | 192 | -12 | -27 | -30 |
| MLT | 233 | 159 | -11 | -10 | -22 |
|  | 233 | 174 | -11 | -18 | -30 |
| Indole | 118 | 91 | -21 | -12 | -17 |
|  | 118 | 65 | -14 | -20 | -27 |
| ILA | 206 | 160 | -11 | -20 | -26 |
|  | 206 | 130 | -10 | -9 | -20 |
| KA | 190 | 162 | -12 | -17 | -11 |
|  | 190 | 144 | -25 | -13 | -28 |
| IAld | 146 | 118 | -12 | -24 | -17 |
|  | 146 | 91 | -13 | -32 | -25 |
| l-dopa | 198 | 152 | -23 | -12 | -30 |
|  | 198 | 181 | -10 | -27 | -24 |
| XAN | 206 | 178 | -21 | -16 | -11 |
|  | 206 | 160 | -10 | -16 | -28 |
| NAS | 219 | 115 | -17 | -17 | -21 |
| QA | 168 | 124 | -11 | -27 | -16 |
|  | 168 | 106 | -10 | -13 | -29 |
| 3OH-KYN | 225 | 162 | -10 | -11 | -20 |
|  | 225 | 152 | -22 | -20 | -19 |
| IPYA | 202.2 | 159 | -23 | -17 | -29 |
|  | 202.2 | 142.8 | -11 | -50 | -22 |
| IA | 186 | 142 |  |  |  |
|  | 186 | 116 | -18 | -12 | -21 |

***** Trp: Tryptophan, IAA: Indole Acetic Acid, TA: Tryptamine, QA: Quinolinic Acid, IPA: Indole-3-Propionic acid, 5-HIAA: 5-Hydroxyindole-3-Acetic Acid, IAM: Indole-3-Acetamide, TRO: Tryptophol, 3HAA: 3-Hydroxykynurenine, 5-HTP: 5-Hydroxytryptophan, 5-HT: Serotonin, KYN: Kynurenine, MLT:Melatonin, ILA: Indole-3-lactic Acid, KA: Kynurenic Acid, IAld: indole-3-aldehyde, XAN: Xanthurenic Acid, NAS: N-acetyserotonin, QA: Quinolinic Acid, 3OH-KYN: 3-Hydroxynurenine, IPYA: Indole-3-Pyruvate, IA: Indole Acrylic Acid
